# Role of Dyadic Proteins in Proper Heart Function and Disease

**DOI:** 10.3390/ijms26157478

**Published:** 2025-08-02

**Authors:** Carter Liou, Michael T. Chin

**Affiliations:** 1 Molecular Cardiology Research Institute, Tufts Medical Center, Boston, MA 02111, USA; carter.liou@tuftsmedicalcenter.org; 2 Graduate School of Biomedical Sciences, Tufts University School of Medicine, Boston, MA 02111, USA

**Keywords:** CMYA5, dyad, transverse-tubule, junctional sarcoplasmic reticulum, junctophilin-2, hypertrophic cardiomyopathy, ryanodine receptor 2, adeno-associated virus, gene therapy, tissue engineering

## Abstract

Cardiovascular disease encompasses a wide group of conditions that affect the heart and blood vessels. Of these diseases, cardiomyopathies and arrhythmias specifically have been well-studied in their relationship to cardiac dyads, nanoscopic structures that connect electrical signals to muscle contraction. The proper development and positioning of dyads is essential in excitation–contraction (EC) coupling and, thus, beating of the heart. Three proteins, namely CMYA5, JPH2, and BIN1, are responsible for maintaining the dyadic cleft between the T-tubule and junctional sarcoplasmic reticulum (jSR). Various other dyadic proteins play integral roles in the primary function of the dyad—translating a propagating action potential (AP) into a myocardial contraction. Ca^2+^, a secondary messenger in this process, acts as an allosteric activator of the sarcomere, and its cytoplasmic concentration is regulated by the dyad. Loss-of-function mutations have been shown to result in cardiomyopathies and arrhythmias. Adeno-associated virus (AAV) gene therapy with dyad components can rescue dyadic dysfunction, which results in cardiomyopathies and arrhythmias. Overall, the dyad and its components serve as essential mediators of calcium homeostasis and excitation–contraction coupling in the mammalian heart and, when dysfunctional, result in significant cardiac dysfunction, arrhythmias, morbidity, and mortality.

## 1. Introduction

The beating of the heart occurs in a temporally and spatially ordered fashion through the propagation of electrical signals in a functional syncytium that triggers synchronized contraction. Action potentials that propagate along the sarcolemma are ultimately converted into the shortening of each sarcomere unit, resulting in muscle contraction. This process is well-documented and is known as excitation–contraction coupling (EC coupling). The cardiac dyad acts as a critical component of this process. Cardiac dyads, as they are so named, consist of two important anatomical parts of the cardiomyocyte, namely the transverse (T) tubule and the junctional sarcoplasmic reticulum (jSR). Together, these two structures allow for the coordinated release of the secondary messenger Ca^2+^. T-tubules are invaginations of the sarcolemma that penetrate deep into each cardiomyocyte. They allow for the action potential (AP) to propagate into the cell, which activates receptors known as L-type calcium channels (LTCC) on their surface, leading to Ca^2+^ influx. As calcium enters the cell, it crosses the space between the T-tubule and the jSR, known as the dyadic cleft. Ca^2+^ that crosses this cleft acts as an allosteric activator of the ryanodine receptor 2 (RYR2) embedded within the membrane of the jSR. The interaction between Ca^2+^ and RYR2 allows for the release of copious amounts of Ca^2+^ from the sarcoplasmic reticulum (SR) into the sarcoplasm in a process known as calcium-induced calcium release (CICR).

Ca^2+^ is an essential ion, as it regulates the cross-bridge cycle within the sarcomere. Each cardiomyocyte is made up of many sarcomeres, which in turn are composed primarily of two proteins, namely actin (thin filament) and myosin (thick filament). The border of each sarcomere is lined by a z-disc, which attaches to the thin filament. The protein titin binds to the z-disc and connects it to the thick filament. The process of cross-bridge cycling occurs as the head of myosin pulls the actin filament towards the M-line, thus shortening the length of each sarcomere. Given the endergonic nature of this process, cross-bridge cycling is tightly regulated to ensure efficient usage of ATP, as well as coordinated contractions of the myocardium. The troponin complex acts as the primary regulator, consisting of three proteins: troponin I, troponin C, and troponin T. Ca^2+^ has a strong affinity for troponin C, which, when bound, leads to a conformational change in the troponin complex, dislodging troponin I and allowing for the myosin heads to bind to actin. Thus, Ca^2+^ is essential to the coordinated beating of the heart, highlighting the structural and anatomical importance of the dyad, which spatially orients the T-tubules and jSR in close proximity to facilitate EC coupling.

It is well-documented that defects in EC coupling and dyadic architecture can lead to heart disease and heart failure [1]. Here, we will focus primarily on arrhythmias and cardiomyopathies. Arrhythmias are defined by abnormal electrical activity, resulting in abnormal beating of the heart. Examples of arrhythmias include tachycardia (a fast heart rate), bradycardia (a slow heart rate), and fibrillation, which presents as random, unsynchronized contractions of the heart muscle and appears as unsynchronized waves on an ECG.

There are three primary types of cardiomyopathies, namely restrictive, hypertrophic, and dilated. Restrictive cardiomyopathies are characterized by a myocardium that is stiff, preventing proper filling of the ventricles. Hypertrophic cardiomyopathies are characterized by a thickening of the myocardium (typically in the ventricle walls), decreasing the efficiency of blood flow through the heart. Dilated cardiomyopathies occur when the ventricular chambers expand, sometimes associated with thinning walls. Overall, cardiomyopathies remain prevalent, with 1 in 500 individuals affected by hypertrophic cardiomyopathy and 1 in 2500 affected by dilated cardiomyopathy globally [2,3]. Oftentimes, cardiomyopathies are inherited, which has presented the opportunity to better uncover their genetic and biochemical mechanisms.

This article will analyze how loss-of-function mutations in various dyadic proteins can manifest as both cardiomyopathies and arrhythmias. It will conclude by discussing adeno-associated virus (AAV) gene therapy that can target diseases in which dyadic proteins are dysfunctional, highlighting the progress made in these therapies and their potential for treating cardiovascular disease.

## 2. The Dyad

### 2.1. Structural Proteins of the Dyad

Dyads are tethered to the z-disc, which serves as the anchor point for sarcomeres, thus maintaining close proximity to sarcomeres and allowing for rapid local calcium release to facilitate EC coupling. The tethering of the dyads to the z-disc is accomplished by three proteins. Cardiomyopathy-associated protein 5 (CMYA5) or myospryn is a large protein of about 4000 amino acids (AA) and is expressed in both heart and skeletal muscle. It is a member of the tripartite motif-containing super-family (TRIM) with the additional protein interaction domains: RING, BBox1 and 2, and coiled-coiled. CMYA5 is highly conserved at both its N terminal and C terminal, and the latter contains a TRIM domain (protein–protein interaction domain). The C terminal of CMYA5 is hypothesized to interact with the sarcomere, specifically with α-actinin 2 (ACTN2), a z-disc protein. However, more research must be conducted to confirm this theory [4].

Through a *Cmya5* KO mouse line, it was shown that ventricular cardiomyocytes had both disorganized jSR and T-tubules. It was also confirmed that CMYA5 organizes the jSR independent of the T-tubule, as atrial cardiomyocytes in *Cmya5* KO mice also lacked structured jSRs. This finding is consistent with other studies showing that T-tubule formation occurs through direct interactions with the jSR and not the z-disc [4].

During dyad formation, the parallel association of the T-tubules and jSRs is known as the junctional membrane complex (jMC). In this complex, LTCC is situated juxtaposed to RYR2 for proper CICR. The protein that allows for this process to occur has been identified as Junctophilin-2 (JPH2), which contributes to T-tubule remodeling, as well as maintaining a stable distance between the T-tubule and the jSR, contributing to jMC formation [5,6]. JPH2 belongs to the JPH family, consisting of four proteins expressed in different tissues that all stabilize the physical distance between the plasma membrane and the SR/ER. JPH1 plays a similar role in skeletal muscles, whereas JPH3 and 4 are expressed in neuronal cisternae [7]. Like CMYA5, JPH2 is highly conserved, specifically at its N-terminus, which contains eight MORN (membrane occupation and recognition nexus) domains that allow for binding to the plasma membrane. Its C terminus acts as an anchor in the SR [7]. Through these two important interactions involving each one of its terminals, JPH2 can recruit LTCC, help structure the T-tubule, and modulate the diameter of the jMC. JPH2′s role was elucidated through its knockdown in animal models, which showed a decrease in the number of jMCs, a disruption in T-tubule shape, and an impairment in the maturation of T-tubules. These results were achieved through di-8-ANEPPS staining, a molecular probe sensitive to electrical changes in the cardiomyocytes [8].

JPH2 extends throughout the dyadic cleft and, thus, holds together the dyad. While it determines where the T-tubule will be generated in relation to the jSR, JPH2 itself is not responsible for T-tubule formation. The bridging integrator protein (BIN1) is part of the Bin–Amphiphysin–Rvs (BAR) protein family, which is involved in membrane curvature. The structure of BIN1 consists of three domains [9]. The first domain, the N-BAR domain located at the N terminal, is a crescent-shaped dimer that has a high affinity for curved and negatively charged membranes, making it an essential part of T-tubule formation [10]. The second domain, the clathrin/AP-2-binding (CLAP) domain, interacts with clathrin, a protein necessary for vesicle formation. The final domain, the SRC Homology 3 (SH3) domain, is involved in cell signaling and transduction pathways [11]. BIN1 is hypothesized to directly interact with JPH2, but it is not well-understood how.

To summarize, the formation of the dyads occurs in a stepwise process involving three key proteins. The z-disc, which contains α-actinin 2, serves as an anchoring point for the C terminal of CMYA5. The CMYA5 N terminal extends and latches to RYR2, tethering the jSR to the z-disc. JPH2 is also attached to the jSR anchored by its C terminal. When the jSR has been correctly positioned by CMYA5, JPH2 then reads the position of the jSR, and BIN1 forms the T-tubule.

### 2.2. Functional Dyadic Proteins

While CMYA5, JPH2, and BIN1 have been discussed extensively in the context of dyadic development, they make up only a fraction of the proteins that are necessary for the dyads to allow for EC coupling to occur. Within the T-tubule, various proteins exist that allow it to carry out its function.

The first protein is the LTCC, which opens in response to depolarization, allowing Ca^2+^ to flow into the cytosol to begin CICR. The LTCC consists of 5 subunits: α1, α2, β, δ, and γ [12]. It is the α1 subunit that forms the pore in the T-tubule membrane and allows for Ca^2+^ influx. The α1 subunit is a monomer that consists of four domains (alpha helices), each made up of six transmembrane segments (S1–S6). Segments 1 through 4 give LTCC the ability to respond to a depolarization specifically coming from signals sent by pacemaker cells [13]. LTCC’s ability to interpret APs is primarily due to the negatively charged amino acids within the S1–S3 segments. Furthermore, four different genes encode for the α1 subunit, which ultimately gives rise to four different isoforms of LTCC. In cardiomyocytes, the isoform Cav1.2 predominates. The α2, β, δ, and γ subunits help to stabilize the α1 subunit through non-covalent bonding [12]. The α1 subunit is believed to be exclusive to the LTCC channels present in muscle cells (Cav1.1 and Cav1.2), and its function has yet to be completely understood [14]. The mechanism behind the activation of the LTCC involves the S4 helix. Upon depolarization of the surrounding membrane, the S1–S4 segments respond, leading to a conformational change in which the S4 helix moves towards the S5–S6 helices. This movement opens an activation gate, allowing for Ca^2+^ to flow into the cell [15].

Regulation of LTCC activity occurs through multiple paths. One is the Ca^2+^/CaM path, which occurs through Calmodulin (CaM), a Ca^2+^-sensing protein that contains EF-hands at its N and C terminus, allowing for the binding of four Ca^2+^ ions at both low and high [Ca^2+^]. Its strong affinity for Ca^2+^ at low concentrations allows it to prevent Ca^2+^ influx into the cell, even when such Ca^2+^ would flow naturally down its concentration gradient [15]. The Ca^2+^/CaM complex inhibits the LTCC through a process known as calcium-dependent inactivation by binding to the IQ domain of the α1 subunit. This leads to a conformational change and closing of the LTCC [16]. It is also well-known that the LTCC is sensitive to 1,4-dihydropyridine (DHP), an organic compound that can act as both an agonist and antagonist through ligand binding to the extracellular side of the α1 subunit [17]. DHP has been widely used as a base for drug development, specifically for regulating LTCC activity.

The calcium that enters the dyadic cleft through the LTCC is responsible for activating RYR2 by binding to the cytosolic portion of the receptor. Named after the diterpenoid ryanodine for which it has a high affinity, RYR2 belongs to the ryanodine receptor family, consisting of three isoforms that regulate calcium release. RYR2 is a large calcium channel that is almost 20,000 AA in size. The shape of RYR2 resembles that of a mushroom with the head protruding from the membrane of the SR. It is homotetrameric, meaning each of the four monomers comes together to form the ion-conducting pore. A closer look at each monomer reveals that they each consist of 10 domains. The first is the N terminal domain, located in the cytosol and involved in protein–protein interactions. Three SPRY domains, which are formed predominantly of β-sheets, act as binding spots for regulatory proteins and are interrupted by the RYR1-2 domain (so named because such domains are found only in ryanodine receptors). RYR2 contains four RYR domains, with one and two and three and four pairing together, respectively. The RYR1-2 domain consists of two α-helices and a C terminal strand. The RYR3-4 contains a phosphorylation loop for cell signaling and interrupts an α-helical domain. The handle and central domain contribute to the gating of the receptor and, thus, increase open probability ($P_{0}$). RYR2 has one transmembrane channel through which the [Ca^2+^] flows out of the cell. Finally, the C terminal domain is involved in the binding of key regulators, namely [Ca^2+^] ATP and caffeine [18].

The regulators of RYR2 work in differing ways. [Ca^2+^] in low doses is known to be a strong activator, helping to explain the mechanism of CICR. In high doses, Ca^2+^ acts as an inhibitor of RYR2, helping to prevent excess muscle contractions. Furthermore, Mg^2+^ acts as a competitive antagonist for the Ca^2+^ binding spot (A spot) on RYR2 [19]. Both ATP and caffeine have been shown to have an agonistic effect on RYR2, increasing its activity. RYR2 can also be phosphorylated by protein kinase A (PKA) and calcium/calmodulin-dependent protein kinase II (CaMKII) in response to sympathetic activity, leading to its activation [20]. Like the LTCC, calmodulin has an inhibitory effect on RYR2 [21]. This makes calmodulin a protein that is essential for the prevention of muscle overstimulation.

Calmodulin, however, is not responsible for Ca^2+^ movement into the SR. That job belongs to the sarco/endoplasmic reticulum ATPase (SERCA). The SERCA family consists of SERCA1, SERCA2, and SERCA3, with SERCA2 found primarily in cardiac muscle. Per ATP hydrolysis, SERCA2 can pump two Ca^2+^ back into the jSR. The importance of SERCA2 is that its activity allows for cardiac muscle relaxation. The structure of the SERCA family has been confirmed through X-ray crystallography. The pump consists of four different domains, three cytosolic and one embedded within the membrane of the SR. Ten transmembrane helices make up the M domain, anchoring SERCA2 into the SR, as well as forming two Ca^2+^ binding domains. For the three cytosolic domains, the N domain or the nucleotide binding site is where ATP hydrolysis occurs. The P domain or phosphorylation domain receives the phosphate group from the ATP hydrolysis reaction, and the A domain or actuator domain signals for conformational changes to pump the Ca^2+^ back into the SR lumen via the M domain [22]. The mechanism behind SERCA’s activity can be explained through the E1/E2 hypothesis [23]. In the E1 state, SERCA’s Ca^2+^ binding sites have a high affinity for Ca^2+^, whereas in the E2 state, the affinity is much lower. The transition between the two states is due to conformational changes dictated by the cytosolic domains and interactions with regulators.

Two key regulators of SERCA2 are the low molecular weight proteins phospholamban (PLN) and sarcolipin (SLN). PLN is a phosphoprotein and a mediator of the adrenergic response in the heart. PLN is an allosteric inhibitor of SERCA2. Through phosphorylation at both serine and threonine residues by protein kinase A (PKA) and CAM-II kinase, PLN becomes dissociated from SERCA2, removing its inhibitory effects [24]. PLN, however, is susceptible to mutations that lead to improper calcium regulation and, thus, cardiomyopathy. For example, the R14del mutation is prominent in the Netherlands and is present in individuals diagnosed with both arrhythmogenic and dilated cardiomyopathy [25]. SLN is a micropeptide that also has an inhibitory effect on SERCA2. It achieves this through two primary mechanisms. The first is through allosteric binding within the transmembrane domain, decreasing the affinity of the Ca^2+^ sites for calcium. The second is more unique, where SLN binding results in the uncoupling of the SERCA. In this process, SERCA continuously hydrolyzes ATP. However, it is unable to efficiently move Ca^2+^ back into the SR lumen [26]. The reason behind this phenomenon is thought to be due to SLN’s ability to cause slippage by disrupting the conformational change needed to push Ca^2+^ back into the lumen [27]. SLN is well-known to play a key role in non-shivering thermogenesis due to its uncoupling abilities. In addition to interacting with SERCA2, SLN has been found to interact with PLN, increasing its binding capacity (shown in the HEK-293T cell culture) [28].

SERCA alone, however, cannot completely relax cardiac muscles. It is aided by the final protein that will be discussed in this section, the sodium–calcium exchanger (NCX), an antiporter protein embedded in the T-tubule that helps to promote calcium efflux from inside cardiomyocytes [29]. The NCX functions by utilizing the electrochemical gradient of sodium, which is much higher outside of the cell. As sodium ions enter the cytosol, energy from that movement is used to move calcium out of the cell, known as the forward mode. Three sodium ions are exchanged per calcium ion [30]. In the reverse mode, calcium is brought into the cell, producing an outward current. The mode of direction of NCX depends primarily on the membrane potential, thus allowing for both depolarization and repolarization of the cell [31].

NCX’s importance lies in its ability to remove excess calcium from the cell and prevent calcium overload. Three NCX genes produce three distinct proteins, with the NCX variant that predominates in cardiomyocytes being called NCX1. The exchanger has been determined to contain a transmembrane domain that consists of 10 transmembrane helices [32]. A large intracellular regulatory domain separates the transmembrane domain into two homologous halves—the first consisting of helices 1-5 (TM1-5) and the second consisting of helices 6-10 (TM-6-10). The transmembrane domain is involved in ion transport, whereas the intracellular domain, which contains two cytosolic calcium-binding sites (CBD), responds to allosteric regulators. CBD1 is thought to contribute to the general structure of NCX1, whereas CBD2 is an activation site. When calcium binds to CBD2 (apo state), there is a conformational change that prevents proper inactivation [33]. The mechanism can be described by the flip-flop theory that involves two conformational states for NCX1 [34]. During the “flip” state, the ion binding sites are exposed, allowing for calcium to bind to the Ca^2+^ binding loop in the transmembrane domain. The conformational change leads to the “flop” state, moving the Ca^2+^ binding loop to the extracellular side and releasing calcium from the cell. At the same time, the “flop” state exposes sodium binding sites within the transmembrane alpha helices, allowing for sodium influx [35]. As calcium levels in the cell drop and sodium levels rise, NCX1 may become inactivated. Inactivation of NCX1 also occurs through the intracellular regulatory domain. The exchanger inhibitory peptide (XIP), which is part of the N-terminus on the cytosolic side of the intracellular regulatory domain, can bind to three sodium ions and lead to inactivation [32]. This prevents excess calcium efflux, as well as excess sodium influx.

Beyond sodium and calcium, the phosphorylation and dephosphorylation of the large intracellular regulatory domain can contribute to the regulation of NCX1. One study has found that muscle-type creatine kinase (CK) was the creatine kinase isozyme that precipitated with NCX1 at the highest rate. It was further found through measuring NCX1 activity in HEK293T cells that, in an energy-compromised state, creatine kinase can maintain NCX1 activity. It is hypothesized that this regulation occurs through a protein kinase c phosphorylation site on CK, and thus, a cycle of autophosphorylation occurs [36]. Additionally, the protein caveolin-3 (CAV3), which acts as a scaffolding protein to help stabilize t-tubules, may contribute to NCX1 regulation [37].

The major dyadic proteins that have been discussed so far, including CMYA5, BIN1, JPH2, RYR2, LTCC, SERCA, and NCX1, while important, do not constitute the entirety of the proteome that forms the dyad. The study concerning CMYA5′s role in dyadic positioning was discovered in 2022, highlighting how novel findings that help paint a clearer picture of EC coupling are still being published. It is important to note that the list of proteins deemed critical to the dyad may change, but as it stands, these proteins are believed to be the most critical and best understood.

### 2.3. Dyadic Protein Interactions

There are multiple well-documented interactions between the major dyadic proteins that have been discussed thus far. The first is the interaction between CMYA5 and RYR2. While CMYA5 has been discussed in its role in the dyadic structure, it also plays an important role in dyadic activity through its regulation of RYR2. As previously mentioned, the terminals of CMYA5 are highly conserved. The C terminal specifically contains a highly conserved region (residues 2731 to 3739) known as MD9, which contains the previously mentioned TRIM domain, as well as an RYR2 binding domain [4]. CMYA5–RYR2 colocalization was confirmed through both a proximity ligation assay (PLA) and co-immunoprecipitation followed by Western blotting. Furthermore, MD9′s ability to interact with RYR2 was proven through AAV, which was used to express the MD9 fragment in vivo. AAV-MD9 was found to co-localize with RYR2 in cardiomyocytes [38].

In terms of physical interaction, it is believed that CMYA5 interacts with the cytosolic region of RYR2, but the exact mechanism remains unknown. What is known, however, is that CMYA5 has an antagonistic effect on RYR2, limiting its ability to release Ca^2+^ and, therefore, may act as a therapeutic target towards harmful arrhythmias. These results were confirmed by generating in vitro single-channel bilayers and measuring the open probability ($P_{0}$) of RYR2. ER (endoplasmic reticulum) vesicles from RYR2-containing HEK293T cells were extracted and then incorporated into the bilayer. The ER vesicles themselves could be manipulated to co-express peptides, such as MD9 and GFP. When introduced, GFP showed no decrease in $P_{0}$. However, MD9 co-expression significantly decreased $P_{0}$, thus confirming the inhibitory effects that CMYA5 has on RYR2. Nevertheless, the biochemical mechanism for MD9 binding to RYR2 and the subsequent response to conformational change, which leads to a decrease in activity, has not been well-elucidated and provides future directions for additional studies.

The jSR acts as somewhat of a mediator in both the dyadic structure and activity, as it interacts with both the t-tubule and the sarcomere. Therefore, since we have discussed CMYA5 and RYR2 in the context of the sarcomere and the jSR, we will now turn towards a vital protein–protein interaction occurring between the jSR and the t-tubule. JPH2 has been discussed as a structural protein that helps to anchor the jSR to the t-tubule. Because an integral part of t-tubule formation and function involves the LTCC, JPH2 has also been found to interact with the channel [5]. The structure of JPH2 consists of six domains: 1. MORN-1; 2. the joining region; 3. MORN-2; 4. a putative helical region; 5. a divergent region, and 6. a spanning region [39].

The MORN domains and the joining regions play a critical role in the interaction between JPH2 and LTCC. MORN, or the membrane occupation and recognition nexus, is known to bind to lipid modules. In JPH2, MORN repeats that are in the N terminal attach to the phospholipids of the t-tubule, helping to orient JPH2 towards the LTCC. The joining region then binds directly to the α1C unit on the LTCC [40]. This leads to the distribution of LTCC throughout the t-tubule, which co-localizes with RYR2. Therefore, this interaction is necessary for proper EC coupling. The results were determined through infecting adult mouse ventricular cardiomyocytes with AAV carrying WT JPH2 or mutant JPH2 that contained seven-point mutations within the joining region. In the latter, it was found that the t-tubule structure was lost, and thus, aberrant calcium signaling was present. Furthermore, it was found that JPH2 was localized to the plasma membrane. A proximity ligation assay (PLA) was used to detect protein–protein interactions, and a patch clamp was used to measure the LTCC calcium currents in AAV-infected cells [39]. The actual mechanisms behind the binding between JPH2 and LTCC, as well as the distribution process throughout the t-tubule, remain largely unstudied.

The function and positioning of the LTCC are not solely influenced by JPH2. Another key t-tubule protein, BIN1, also exercises control over the voltage-gated channel. Together, JPH2 and BIN1 allow for LTCC to play its role in proper CICR. It has been found that BIN1 co-localizes with LTCC and that they cluster together within 10-50 nm of each other in the t-tubule. This data was produced through co-immunoprecipitation through rec protein-G sepharose pulldown. Because LTCC is trafficked to the t-tubule from the Golgi apparatus, efficient transport of LTCC is an important part of dyadic formation. BIN1 has been found to anchor to microtubules, which are required for the delivery of LTCC to the t-tubule. This was confirmed through the BIN1 knockout cardiomyocytes in which LTCC plasma membrane expression was significantly reduced [41].

CMYA5 has been well-documented in its role in the positioning of the jSR. Likewise, BIN1 and JPH2 have been well-documented in their role as the primary proteins that contribute to the depth, shape, and positioning of the t-tubule. Here, we highlight how structural proteins also regulate the activity and organization of other proteins within the dyad. These proteins are, therefore, multifaceted and essential to cardiac function. In the following section, we will discuss the consequences when various dyadic proteins do not function properly.

## 3. Mutations

### Dyadic Mutations

Small mutations in both gene and amino acid sequences can, unfortunately, lead to serious and debilitating conditions. The heart is not spared from this reality. Many cardiovascular conditions that arise during development, and even as we age, are linked to mutations in vital cardiac proteins. Mutations in RYR2 are well-known to be associated with various arrhythmias, specifically catecholaminergic polymorphic ventricular tachycardia (CPVT). CPVT is an autosomal-dominant disease that is characterized by distinct ECG waves. The pattern with which it is associated is known as bidirectional ventricular tachycardia, characterized by alternating positive and negative waves. Furthermore, there is polymorphia in the ventricular waves [42].

CPVT is a deadly disease that can lead to sudden cardiac death. Furthermore, the disease may go undetected, as the heart structure is unremarkable and asymptomatic ECGs are normal under resting conditions. CPVT is, therefore, triggered during times of adrenergic stimulation or physical exercise (normal levels of physiological stress), which has led researchers to believe that mortality rates by the age of 20–30 for those with CPVT are 30% to 50%. RYR2 contains a carboxyl terminal domain that contains the transmembrane segments of the receptor. Over 200 mutations have been identified that link RYR2 to CPVT. Three missense mutations have been described in detail. A proline substituted for a serine (P2328S) mutation was found in RYR2′s footlike cytoplasmic domain. An additional two mutations were found in the carboxyl terminal domain, namely Q4201R and V4653F [42]. Mutations in RYR2 that lead to CPVT result in an increase in diastolic Ca^2+^ due to the sudden increased activity of all RYR2 receptors on the SR [43]. In response to physical activity and stress, catecholamines, such as epinephrine, are released from the adrenal gland and activate β-adrenergic receptors, which in turn lead to phosphorylation of RYR2 by kinases such as PKA. This signaling pathway leads to pore opening. Therefore, mutations in RYR2 lead to abnormal Ca^2+^ release in the form of calcium sparks. A vicious cycle develops, further inducing CICR and calcium waves that manifest on an ECG as afterdepolarizations with the potential to trigger arrhythmias, such as CPVT [44].

There are various treatment options for CPVT. Medical therapy has been the most successful, specifically β-blockers as a first line of defense and then sodium channel blockers, specifically flecainide, for patients where β-blockers are ineffective. Procedural therapies such as cardiac sympathetic denervation have also been utilized. Treatment of CPVT does, however, present unique challenges. For example, for many channelopathies, implantable cardiac defibrillators (ICDs) act as a lifesaving therapy. However, the risk of inappropriate shocks from an ICD precludes it as an option for many with CPVT [45].

Two future ideas for treatment are currently under discussion. The first is through CMYA5′s interaction with RYR2. As previously discussed, CMYA5 contains an MD9–RYR2 interacting domain that, in isolation, can significantly decrease the $P_{0}$ of RYR2, thus acting as a reversible inhibitor. Introducing this peptide fragment as a therapeutic option may hold promise. Additionally, gene therapy has been a topic of exploration. In the context of RYR2, gene therapy has not been studied to correct for missense mutations in the RYR2 sequence but rather to directly target a well-known activator of RYR2, CaMKII. An adeno-associated viral (AAV) vector was generated containing autocamtide-2-related inhibitory peptide (AIP), a potent inhibitor of CaMKII, fused to green fluorescent protein (GFP). Results showed that the introduction of AAV-GFP-AIP to cardiomyocytes derived from CPVT patients suppressed aberrant Ca^2+^ release from the jSR [46].

RYR2 is not the only dyadic protein that can lead to arrhythmias. The Bowditch effect, or the staircase phenomenon, refers to the idea that, as heart rate increases, the force of contraction that the cardiomyocytes generate also increases. This is a normal phenomenon in a healthy heart, termed a “positive staircase,”. However, it may also lead to arrhythmogenesis. SERCA has been found to be a master regulator of the Bowditch effect. In cases where there is a negative staircase, indicative of heart failure, SERCA is often downregulated. Physical mutations in SERCA were hypothesized to change the staircase pattern. Research was conducted looking at two-point mutations. The first, A617T, is believed to disrupt the ATPase cycle in SERCA, and the second, E442K, is thought to interfere with ATP binding, leading to a conformational change in SERCA. Interestingly, when such mutations were introduced in *D. melanogaster*, whose SERCA is highly conserved with humans, the effects on cardiac output differed. A617T resulted in a positive staircase compared to control, whereas E442K resulted in a negative staircase compared to control [47]. In humans, the expected phenotype for A617T would be the risk of arrhythmias, whereas for E442K, there would be potential for heart failure.

The mechanism behind SERCA mutations, heart failure and arrhythmogenesis is likely related to effects on jSR calcium stores and free SR Ca^2+^, which is affected by calsequestrin-2 buffering capacity. Increased interaction of free SR Ca^2+^ with RYR2 Ca^2+^ sensitive sites on its cytosolic domain that increase its open probability thus increase the cytosolic levels of Ca^2+^ and promote arrhythmogenesis [48].

To connect a positive staircase to the risk of arrhythmias, researchers measured the portion of flies that showed ectopic beats, and the researchers also developed an arrhythmicity index defined by heart rate variability. Utilizing a conducting device connected to an electro-stimulator and computer, various frequencies could be administered to try to induce ectopic beats/arrhythmias in the flies. However, with the frequencies chosen (range 1 to 6 Hz), the control groups were unaffected. Flies with the A617T mutation, however, saw a significant increase in both the arrhythmicity index and the number of ectopic beats in response to increased frequency [47].

This demonstrates the possibility that mutations in human SERCA may be connected to arrhythmias. Furthermore, while the E442K mutation, which exhibits a negative staircase effect, did not see a significant difference from control groups in terms of arrhythmicity index and ectopic beat count, it may be linked to various cardiovascular diseases, such as cardiomyopathies and heart failure, given current findings [46]. For these reasons, SERCA has become a target for gene therapy to ensure its role in cardiomyocyte relaxation.

One study has demonstrated the potential of such gene therapy. In the study, a novel AAV9. SERCA2a vector was developed that increased myocardial levels of SERCA2a in rats with heart failure (HF). Additionally, HF in rats was associated with frequent and spontaneous ventricular arrhythmias, which were significantly reduced upon injection of AAV9. SERCA2a, when compared to the GFP control. AAV9. SERCA2a was found to specifically reduce the degree of QT prolongation. The antiarrhythmic properties of this novel gene therapy may be due in part to its stabilizing effects on calcium release. It was determined that, following tetracaine perfusion, a compound that significantly decreases $P_{0}$ SR Ca^2+^ leak was significantly reduced in HF hearts infected with AAV9. SERCA2a compared to control. Finally, it was shown that SERCA gene therapy also had the ability to alter biochemical responses to heart failure. While both RYR2 and PLB phosphorylation were elevated in HF rats, they remained unchanged in the HF rats exposed to AAV9. SERCA2a [49]. The CUPID 2 clinical gene therapy trial examining the utility of SERCA2a gene therapy, however, gave disappointing results [50]. This may be attributed to the specific serotype of AAV used, the existence of antibodies against AAVs, and dosage.

Hypertrophic cardiomyopathy (HCM) is defined by thickening of the myocardium, specifically in the ventricles and septum. This occurs specifically in the absence of any underlying cause, such as hypertension or valvular heart disease. Thickening can reduce the heart’s ability to pump blood efficiently. HCM is a condition of diastolic dysfunction, as the stiff and thickened wall prevents the ventricle from filling properly. HCM tends to develop in early life and has a well-documented prevalence of about 1 in 500 people. HCM can also be associated with arrhythmias and may lead to sudden cardiac arrest. Due to its prevalence, hereditary nature, risk during embryonic development, and potential to be life-threatening, extensive research has been conducted in an effort to better understand HCM. Many forms of HCM are autosomal dominant, and it is historically associated with mutations in sarcomeric proteins, such as myosin binding protein C3 (MYBPC3) [2]. However, mutations in dyadic proteins also play a role in the development of HCM.

The first protein that will be discussed is JPH2. It was found that JPH2 knockdown mice (in a cardiac-restricted manner) prevents the maturation of T-tubules, showing that JPH2 plays an important role in dyadic formation and heart function [8]. Three missense mutations were identified in patients with HCM, namely S1014, Y141H, and S165F. All three mutations were found to be within the MORN domain of JPH2 and were determined to disrupt JPH2′s ability to bind to the plasma membrane (a key part of the protein’s function), as well as be phosphorylated at its C terminal [51].

It is thought that such mutations in JPH2 may lead to vacuolization of the SR, severely disrupting calcium signaling [51]. Ultimately, the connection between JPH2 and HCM lies in the idea that, if calcium signaling is reduced, this causes reduction in sarcomere activity and thus cardiomyocyte contraction. As the cardiomyocytes become more dysfunctional, they respond through hypertrophy to compensate for the inefficient calcium signaling. This compensatory adaptation can damage the cardiomyocyte over time, which is why hearts with HCM characteristically stain positive for collagen in trichrome staining [52]. Because proper calcium regulation is essential to prevent HCM, many other dyadic proteins, such as LTCC and SERCA, may also play a role in HCM.

Treatment for HCM has traditionally involved medications such as β-blockers (antiarrhythmic drugs), changing lifestyle, which includes limiting caffeine and avoiding intense physical activity, and surgical procedures such as myectomy or septal ablation. While proven effective, these options require continuous treatment to prevent relapse [5]. Gene therapy has shown promising results. One study demonstrated how administration of AAV-mediated overexpression of WT JPH2 could prevent heart failure in mice who underwent a transverse aortic constriction (TAC) procedure. A surgery in which the aorta is constricted, TAC forces the heart to pump more forcefully against artificial resistance. This increases blood pressure (pressure overload) and, over time, promotes pathological left ventricular hypertrophy (LVH), which is a hallmark in HCM. It was determined that, following TAC surgery and subsequent AAV9-JPH2 administration, the ejection fraction significantly increased. These results were explained by AAV9-JPH2 enhancing calcium transients, which most likely led to controlled contractions and decreased strain on cardiomyocytes [53]. Therefore, the administration of this form of gene therapy could help improve outcomes for patients diagnosed with HCM connected to mutations in JPH2.

While HCM is the predominant form of cardiomyopathy, other types do exist and are worth highlighting. Dilated cardiomyopathy is defined by dilation of the muscular wall of the heart, typically starting in the left ventricle. Loss of inotropy, or the inability to contract, leads to a decrease in pumping blood out of the ventricles. Thus, while in both HCM and DCM cardiac output (CO) is reduced, DCM is a disease of systolic dysfunction. Unlike HCM, DCM typically develops later in life. However, its prevalence is still a concern at roughly 1 in 2500. Mortality levels remain high for those with DCM due mostly to failure in pumping or sudden cardiac death from arrhythmias. Death rates from the 1990s to the 2010s rose from 5.4 to 5.9 per 100,000 globally, an indication that DCM continues to be a deadly disease [54]. Additional forms of cardiomyopathy exist, such as non-compaction (NCCM) and arrhythmogenic cardiomyopathy (ACM). While the former has not been found to be associated with loss of function in the dyad, ACM has been linked to mutations in RYR2 [55].

Two key dyadic proteins have been found to be associated with DCM. The first is BIN1. Interestingly, when BIN1 is knocked out in the embryonic stage of mice, it leads to HCM. However, KO of BIN1 at the age of 8–10 months induces DCM. Such differences may be due to BIN1′s role in both the structural maintenance of the dyad and influencing Ca^2+^ signaling. In this paper, we will focus, however, on BIN1′s connection to DCM. TAC surgery was performed to induce pressure overload in the left ventricles of mice with nonfunctional BIN1, resulting in an accelerated loss of cardiac function. The naturally occurring BIN1 retained in the absence of Cre recombinase was found to act as a defense against such stressors [56]. Because BIN1 interacts with many proteins, such as MYC, CAV3, and LTCC, it is hypothesized to stabilize the structure of the cell and is, therefore, believed to combat age-related complications in the heart based on its role in preventing age-related dementia. This may also explain why it is associated, long-term, with DCM. Decreased stability in the cardiomyocytes deficient in BIN1 may result in damage from mechanical stretching.

CMYA5, a master protein for dyadic architecture, is also associated with both dilated and ischemic cardiomyopathy, likely for similar reasons. A loss-of-function CMYA5 mouse model was generated by creating a frameshift mutation in exon 2 using a CRISPR/Cas9 system and two guide RNAs. The results revealed an increase in heart weight compared to body weight and a decrease in systolic function, coupled with dilation of the ventricles. Additionally, it was determined that biochemical stress promotes dyadic disorganization, weakening the heart. CMYA5, like BIN1, was found to shield cardiomyocytes through stabilization of the dyad. When pressure overload was induced by TAC, CMYA5 KO mice developed severe ventricular dysfunction compared to WT mice, which remained relatively stable for 4 weeks following the procedure [4].

Currently, there are various well-established treatment options for dilated cardiomyopathy. Non-invasive choices include ACE inhibitors and β-blockers, which represent the standard therapy. Increased physical exercise is also recommended. However, the optimal amount to minimize complications pertaining to DCM remains undetermined. Cardiac resynchronization therapy (CRT), which simultaneously activates the ventricles through electrical activity by a CRT pacemaker, improves heart efficiency in patients with systolic heart failure associated with risk of sudden cardiac death [3]. Due to the poor prognosis of DCM once diagnosed, emphasis has been placed on preventative measures, such as health-conscious habits and active monitoring. Novel findings in gene therapy have shown promise in averting DCM and eliminating symptoms that do arise.

Both BIN1 and CMYA5 have proven to be successful in transduction by AAV by improving the structure and function of the heart. AAV-BIN1 was shown to effectively restore T-tubule/cBIN1 microfolds and, therefore, improve systolic function in mice undergoing TAC [57]. Due to CMYA5′s size, an engineered version named *miniCMYA5* was generated to accommodate the size capacity of AAV. Efforts to generate a viable *miniCMYA5* were undertaken, given AAV’s historic success in efficiently transducing cardiomyocytes [38].

*MiniCMYA5* consists of a *Tnnt2* promoter expressing residues 1 to 329 (N terminal) and a C terminal fragment containing MD9 from the original CMYA5 protein. Due to their immaturity, cardiomyocytes derived from human induced pluripotent stem cells (hiPSC-CM) have low CMYA5 expression. Introduction of AAV-*miniCMYA5* to hiPSC-CMs resulted in alignment of the jSR and, thus, the dyad to the z-disc, which greatly improved control of Ca^2+^ levels. This indicated that the bioengineering was successful and that *miniCMYA5* functioned as a genuine substitute for CMYA5. AAV-*miniCMYA5* was also found to be therapeutic. In WT mice undergoing TAC that were treated with the AAV-control virus, severe pathological hypertrophy developed. This differed from WT mice that underwent TAC and received AAV-*miniCMYA5,* in which there was an increase in fractional shortening, a metric for systolic function [38]. These results for the gene therapy demonstrate the ability to not only treat but also prevent the development of DCM and HCM.

The dyad is a structure that is, in many ways, fragile. Even the slightest change, such as a point mutation in a single protein, can not only disrupt its architecture but also EC coupling, leading to devastating consequences. Extensive research and efforts have been exerted towards understanding dyadic proteins, the diseases they may cause, and developing potential treatments.

## 4. Conclusions

**Figure 1:**
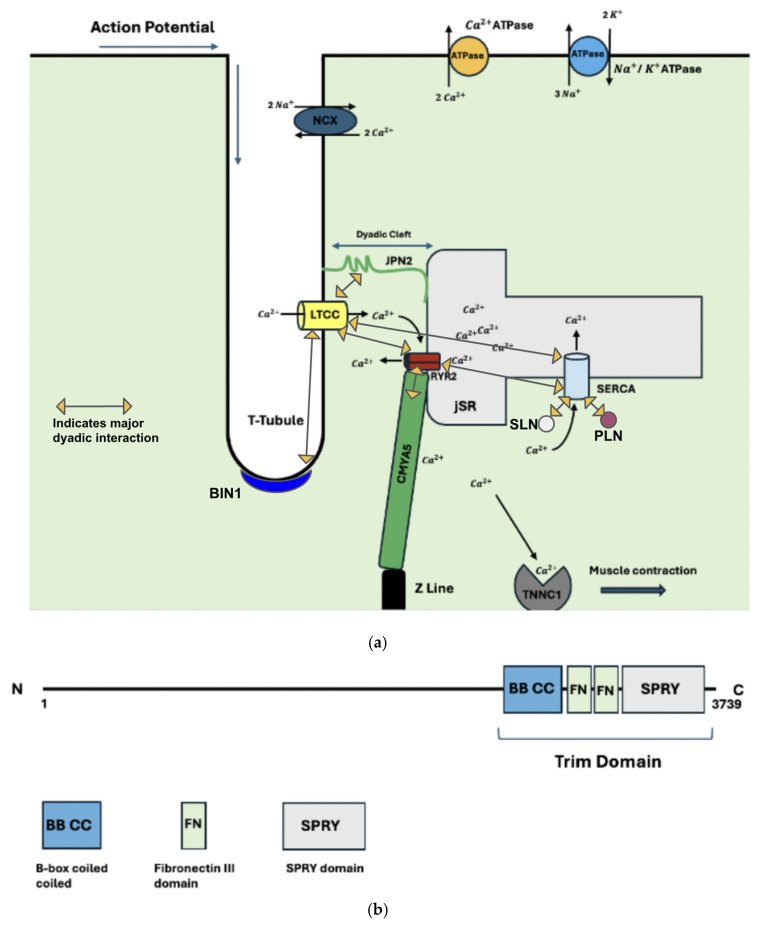
(**a**) Anatomical organization of the dyad. Included are key structural proteins. The process of EC coupling is highlighted, as the direction of the action potential, along with the corresponding movement of calcium throughout the cardiomyocyte, is included. (**b**) CMYA5 schematic. Both N and C terminals are shown. The N terminal interacts with the z-disc, whereas the C terminal interacts with RYR2, anchoring the dyad to the sarcomere. TRIM protein binding domain and its components are included.

The dyad is an essential component of the heart, and its structure is shown in Figure 1. The dyad connects the depolarization of the sarcolemma to the contraction of the sarcomere through the transportation of Ca^2+^. For this process to occur efficiently, dyadic proteins must not only be structurally sound but also positioned correctly. Proteins such as LTCC, RYR2, SERCA, and NCX move Ca^2+^ throughout the cardiomyocyte, regulating its sarcoplasmic concentration and, thus, the activity of the sarcomere. Proteins such as BIN1, JPH2, and CMYA5 are responsible for the structural integrity of the dyad, as well as the positioning of the dyadic transporters.

Mutations in dyadic proteins not only affect the dyad structure and function but, additionally, cause global dysfunction of the whole heart. Arrhythmogenic disorders, such as CPVT, and cardiomyopathies, such as HCM, are shown to be caused by defects in dyad structure and function. Given the many complications associated with diseases such as CPVT and HCM, effective treatments, such as gene therapy, are needed to improve the lives of affected patients.
